# New simplified criteria for predicting massive transfusion in trauma

**DOI:** 10.1186/cc13299

**Published:** 2014-03-17

**Authors:** T Yumoto, A Iida, E Knaup, N Nosaka, S Morisada, T Hirayama, N Shiba, K Tsukahara, H Nosaka, Y Kinami, M Terado, H Yamanouchi, K Sato, T Ugawa, S Ichiba, Y Ujike

**Affiliations:** 1 Okayama University Hospital, Okayama, Japan

## Introduction

Several predicting models have been described to identify the necessity for massive transfusion (MT) for trauma patients [1]. The purpose of this study is to validate the simplified scoring systems reported previously and establish new criteria at emergency and in the ICU in Japan.

## Methods

We retrospectively analyzed trauma patients transported to our center for the recent 2 years. Patients transferred from other hospitals with minor injuries or confirmed cardiac arrest at the scene were excluded.

## Results

A total of 297 trauma patients were included in this study. Thirty-one (10.4%) patients required MT. Sensitivity and specificity for the Assessment of Blood Consumption (ABC) score were 48% and 99%, respectively. Because blunt injuries account for most trauma patients in Japan, we established new simple criteria using significant factors that were derived from the examination on arrival. If trauma patients met any of the following conditions - that is, shock index (SI) >1, base excess (BE) <-3 mmol/l, and positive focused assessment of sonography for trauma (FAST) - sensitivity and specificity was 97% and 80%, respectively. The area under the receiver operating characteristic curve of ABC and the new criteria was 0.889 (95% CI, 0.815 to 0.963) and 0.927 (95% CI, 0.881 to 0.974), respectively.

## Conclusion

We suggest new criteria for early predicting MT in trauma using SI, BE, and FAST. This method may be valid especially in such areas because blunt injuries are the major cause of trauma in Japan.
